# Facilitating endoscopic submucosal dissection: double balloon endolumenal platform significantly improves dissection time compared with conventional technique (with video)

**DOI:** 10.1007/s00464-018-6336-4

**Published:** 2018-07-16

**Authors:** Sam Sharma, Kota Momose, Hisashi Hara, James East, Kazuki Sumiyama, Kiyokazu Nakajima, Gerd Silbehumer, Jeffrey Milsom

**Affiliations:** 1 Weill Cornell Medicine, New York Presbyterian Hospital, 1300 York Ave, New York, NY 10065 USA; 2 Oxford Radcliffe Hospital, Oxford, OX3 9DU UK; 3 Jikei University School of Medicine, Tokyo, Japan; 4 Osaka University, Osaka, Japan; 5 Medical University Vienna, AKH-Wien, Vienna, Austria; 6 MINT, 641 Lexington Ave, Fl 25, New York, NY 10065 USA

**Keywords:** Endoscopic, Submucosal, Dissection, Retraction, Double balloon

## Abstract

**Background:**

Flexible endoscopes ability to manipulate the intestinal environment is limited. As a result, complex endolumenal procedures are often technically demanding and result in long procedure times, impacting institutional resources. Single- and double-balloon add-on endoscopic devices have been employed throughout the GI tract to facilitate tissue control e.g., small bowel enteroscopy, with recent reports suggesting a possible colonic utility for complex procedures e.g., ESD. Our objective was to objectively analyze the efficacy of a new double-balloon device in performing ESD.

**Methods:**

*Ex vivo*—12 simulated colonic lesions were created in porcine rectum using a standard 40 mm diameter template. Two categories were evaluated, standard cap technique ESD and double-balloon assisted ESD with retraction (ESD-R). Cases were performed sequentially. In vivo*—*Six, 40 mm lesion ESD-R’s were performed in a porcine model. The primary outcomes of this study were total procedure and dissection times.

**Results:**

In *ex vivo* studies, the median total procedure time with the double-balloon platform was significantly shorter than the traditional ESD technique (29 ± 18 vs. 57 ± 21 min, *p* = 0.03). In the in vivo studies, lesions were successfully removed in a mean time of 48 min, with a dissection time of 20 min with no significant complications. Balloon-clip retraction and specimen retrieval capabilities were used in all double-balloon assisted cases. After 6 cases, times were significantly shorter (*ex vivo* 47 vs. 17 min; in vivo 57 vs. 27 min).

**Conclusions:**

We have demonstrated the development of a unique technical ESD method facilitated by a new double-balloon device. *Ex* and in vivo investigation demonstrated superiority of ESD-R over the conventional *ex vivo* method. The DB device provided increased stability, improved visualization and tissue traction, which significantly reduced dissection time. Such an approach may increase safety, improve patient outcomes, and may prevent unnecessary surgeries for benign conditions.

**Electronic supplementary material:**

The online version of this article (10.1007/s00464-018-6336-4) contains supplementary material, which is available to authorized users.

Colorectal cancers are currently amongst the most common worldwide, with over 136,000 new diagnoses and almost 52,000 deaths in the United States alone [1]. For complex benign polyps (the colon cancer precursor), advanced procedures facilitated by flexible endoscope (FE) technology permits organ-preserving lesion removal, and the prevention of unnecessary surgical intestinal resection. Examples of advanced techniques facilitated by FE technology are endoscopic mucosal resection (EMR), endoscopic submucosal dissection (ESD), and hybrid procedures [2]. FE design facilitates intestinal navigation, however, the technology remains inherently unstable, particularly with regards to right sided intestinal lesion management and redundant intestinal segments. In addition, the ability to fully visualize the mucosa in and around folds is limited due to the camera’s fixed position. In procedures such as EMR and ESD, multiple challenges [3–6] often exist such as lack of FE stability and poor mucosal visualization. As a result, the procedural technical difficulty increases. A significant component of this challenge may be attributed to the lack of effective tissue traction that allows for ESD to be performed safely and reduce procedural technical complexity. In traditional surgeries, assistants provide continuous effective traction; this is currently difficult in traditional endoscopic procedures. In addition, endoscopic specimen retrieval can be challenging since it requires a variety of tools and use of the working channel, or removing the scope in its entirety [7–9].

The primary objective of this study was to assess the potential advantages of a double-balloon device in performing complex endolumenal procedures such as ESD. The second was to evaluate whether a novel ESD technique development in an *ex vivo* model could be readily transferred in vivo.

## Materials and methods

### Double-balloon endolumenal intervention platform (DEIP)

#### DEIP description

**Fig. 1:**
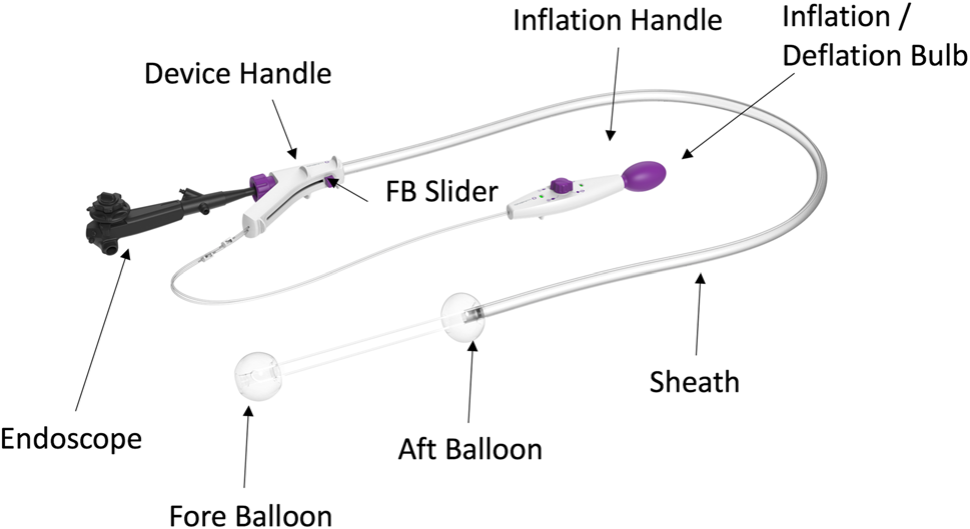
DEIP overview

**Fig. 2:**
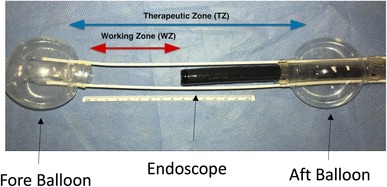
DEIP device balloon configuration

A U.S. Food and Drug Administration–approved commercially available DEIP (DiLumen™, Lumendi, Westport, CT) was used for all cases (Fig. 1). The DEIP comprises a 168 cm flexible polyurethane oversheath with two independently inflatable balloons. The Aft-Balloon (AB) sits behind the endoscope tip and is fixed in position whereas the Fore-Balloon (FB) can be moved beyond the endoscope tip to an operator defined distance. With the FB extended beyond the FE tip and both balloons inflated this was termed the therapeutic zone (TZ) (see Fig. 2). The FE was passed through the sheath using water or gel based lubricant to ease passage either by irrigating the internal surface of the sheath whilst passing the FE or liberally applying gel lubrication liberally on the outer surface of the FE upon insertion. Once passed through the sheath with the FE tip 1 cm proud of the FB the FE was locked at the device handle (see Fig. 1—purple knob on device handle into which FE is inserted) using an incorporated circular silicone clamp (Tuohy Borst valve) to prevent device movement within the sheath. The device handle was then connected to an inflation handle (see Fig. 1), permitting individual inflation or deflation of the selected balloon using the inflation/deflation bulb (see Fig. 1). Both balloons reach a fully inflated diameter of 6 cm with an internal pressure of 55 mmHg. The device was equipped with an over-inflation safety valve which ensured the internal pressure of the balloons did not exceed 55 mmHg. The AB provided endoscope tip stability, whereas the adjustable FB provided mucosal gripping, the ability to flatten folds, straighten flexures, and provide tissue retraction.

#### DEIP use

The device can be used with the subject positioned in lithotomy or in left lateral orientation. After per rectum examination, the DEIP was mounted on the FE and both inserted into the anus after lubrication. FE functions occur as normal including the articulating section.

#### Setting up the TZ

At an appropriate section of the intestine, the AB inflation selector was selected via the inflation handle control knob on the inflation handle. The inflation/deflation bulb was squeezed until the desired pressure was reached (indicated by a constant green indicator). Upon confirmation of scope stability using longitudinal movements on the scope shaft, the FB was extended beyond the endoscope tip using the FB slider located on the handle of the device (see Fig. 1). Upon extension to the desired distance the FB inflation position was selected and the inflation bulb was again squeezed to inflate the FB, the degree of which was confirmed using the green indicator as well as visual representation on the endoscopic view. After confirmation of mucosal gripping, the FB was further extended using the handle knob to provide mucosal traction. Following the procedure, the reverse of the balloon inflation was performed and the FB redocked onto the endoscope tip.

### *Ex vivo* study

**Fig. 3:**
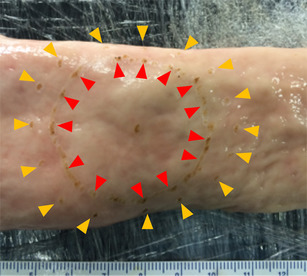
Inverted porcine rectum with lesion (red arrows) and 5 mm margin (yellow arrows) demarcated with electrosurgery

**Fig. 4:**
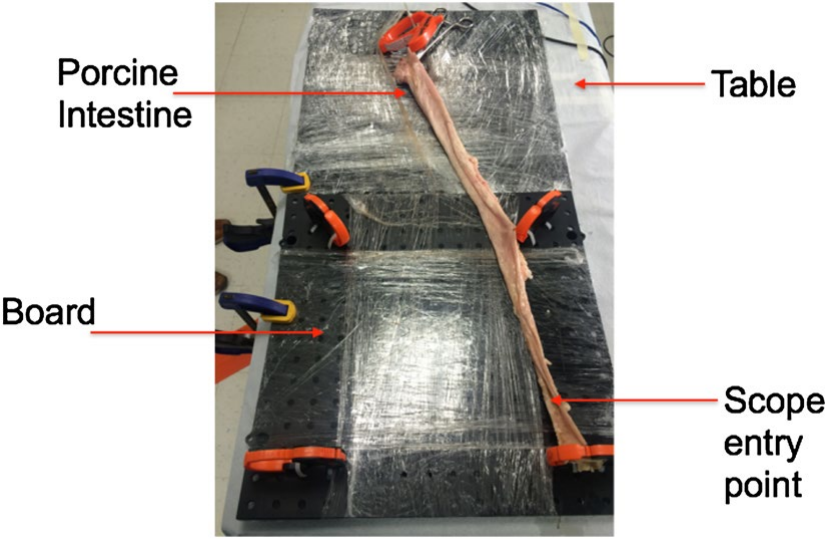
ESD model

Using fresh *ex vivo* porcine rectum, 4 cm “polyps” (with 5 mm margin) were created using electrosurgery (see Fig. 3) and positioned within an established ESD model (see Fig. 4).

Two different polypectomy methods were evaluated and compared:

1. ESD with retraction (ESD-R)—*n* = 6
2. Traditional cap-assisted ESD technique—*n* = 6 (*ex vivo* only)

### In vivo study

**Fig. 5:**
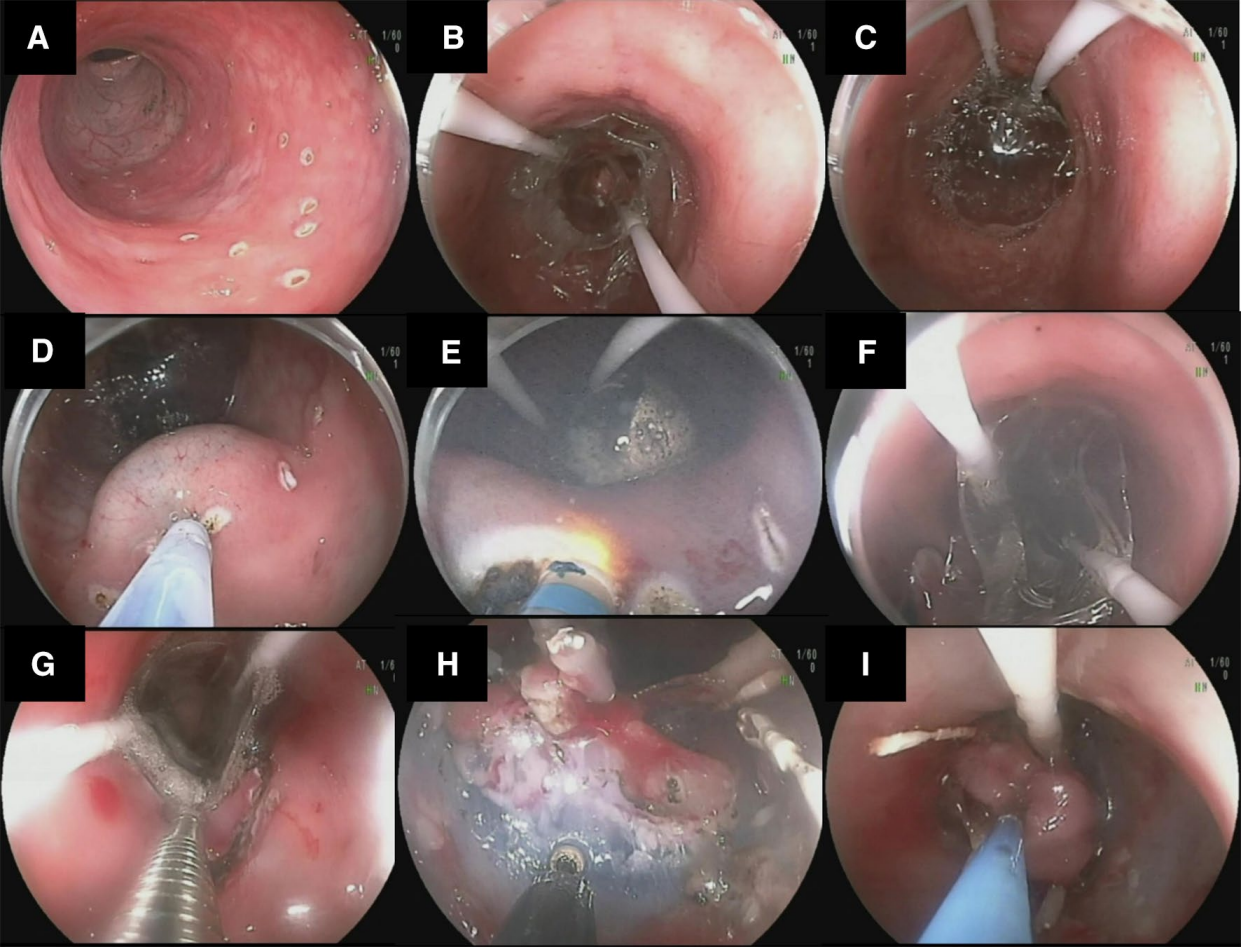
ESD-R technique

All animals studies were sanctioned according to the Japanese IACUC guidelines and Osaka University Animal Research Committee. A 55 kg Yorkshire pig was anesthetized with 5% isoflurane and monitored throughout. The first 50 cm from the anus were used for the experiments as proximal to this level the intestine thinned significantly due to transition to the porcine spiral colon. After careful lavage, simulated intestinal polyps were created by using marking dots with an ESD electrosurgical knife with 1 cm markings to 40 mm in diameter (Fig. 5A).

#### ESD-R technique (see Video 1 and Fig. 5)

First, the balloons were deployed proximal and distal to the lesion (see Fig. 2). A circumferential mucosal incision was made at the lesion margin. The leading edge (closest to the endoscope tip) was then developed further. Once completed, the mucosal edge was clipped to the base of the fore-balloon (see below). Using variable tension on the fore-balloon, tissue dissection continued until resection completion. Knife use was standardized across all procedures. The Dual-knife was used to facilitate entry to the proximal border edge of the lesion, after which the knife was changed to the IT-nano and used throughout till dissection completion.

#### FB-clip retraction

Hemostatic clips (Long Clip, HX-610-090L; Olympus Medical Systems Corp., Tokyo, Japan) were placed in 2–3 places connecting the developed leading mucosal edge to the specially designed shelf of the FB (see Fig. 3F–H). Upon extending out the FB using the FB slider (see Fig. 1), the leading edge of the polyp was retracted in the opposite direction of the endoscope tip providing an unobstructed view with significant traction.

#### FB specimen retrieval

Post FB-clip retraction, the specimen was directly attached to the FB, following this, the free edge of the lesion was grasped and placed into the FB central channel. If necessary the FB was inflated slightly to close the lumen and facilitate removal.

#### Equipment

UESD-R (DiLumen™, Lumendi, Westport, CT) or traditional cap-assisted ESD method (Olympus cap D-201-12704) was performed using a pediatric colonoscope (Olympus PCF-H180AL). Monopolar electrosurgery using ERBE electrosurgical generator with Olympus Dualknife (KD-650U) and IT-nano (KD-612U), 80w Cut 40w Coagulation. The same current settings were used for all parts of the procedure (Autocut and swift coag). Submucosal injection was used in all cases (0.04% methylene blue, normal saline solution) through a Boston Scientific 25G endoscopic needle injector.

Multiple endoscopists experienced in advanced endoscopic actions (S.S, J.E, K.S, G.S. J.M) performed all in vivo procedures. *Ex vivo* ESD was performed by two endoscopists (S.S and J.M) in a sequential alternate fashion (i.e., SS cap, JM cap, SS ESD-R, JM ESD-R…).

Procedural times were recorded. Time to perform the circumferential incision and submucosal dissection were recorded. Occurrence of perforations was recorded. Minor perforations were those deemed to have no breached the serosal layer. Major perforations were those deemed to have breeched the serosa. The maximal diameter of the specimen as well as % completed was determined after resection by one designated individual (SS). Video and photo were taken of all procedures.

### Definition of outcomes

The primary outcome measurement of this study was total procedure time and dissection time. Secondary outcome measurements included occurrence of adverse events such as perforation.

### Statistical analysis

The Students *t* test was used to compare total procedure time. Data are shown as median with standard deviation (SD). All statistical analyses were performed by using GraphPad Prism (GPSoftware Inc, UKR), with results considered significant if *p*< 0.05.

## Results (see Table 1)

### *Ex vivo* ESD

**Fig. 6:**
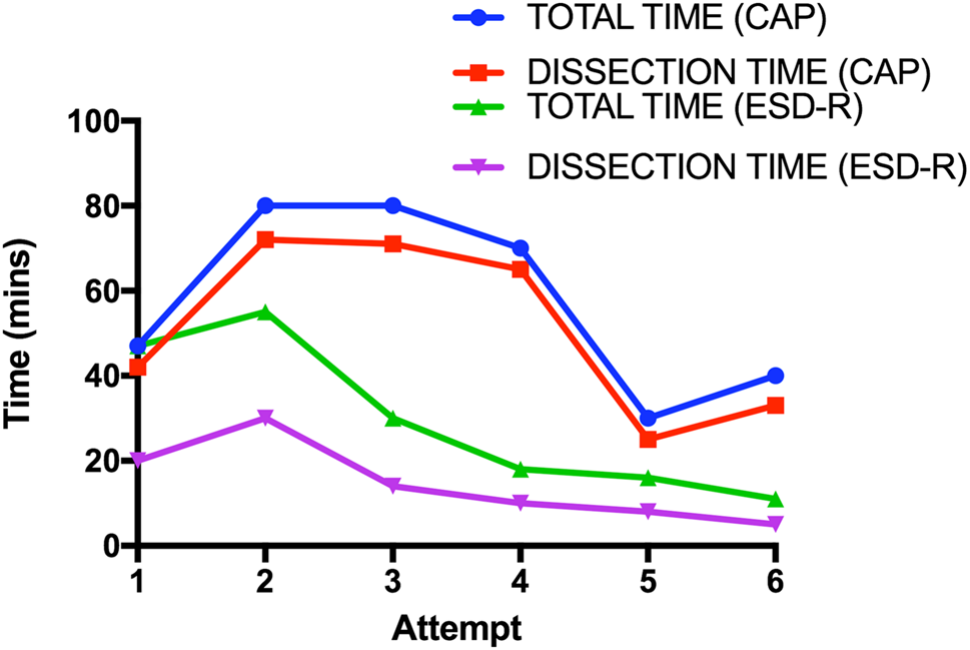
Time by attempt (cap vs. ESD-R technique)

FB-clip placements were successful in all six cases. Median time for ESD-R was 29 min (± 18), which was almost half the time taken with the cap-assisted ESD (57 ± 21 min). This time reduction was statistically significant (see Fig. 6, *p* = 0.03). There was 1 minor perforation in the ESD-R group (attempt 4).

**Table 1:** *Ex vivo* and in vivo procedural results

Experiment number	Ex/in vivo	Operator/assistant	Procedure	Total time (min)	Dissection time (min)	Minor perforations
1	*Ex vivo*	SS/JM	CAP	47	42	0
2	*Ex vivo*	JM/SS	CAP	80	72	0
3	*Ex vivo*	SS/JM	ESD-R	47	20	0
4	*Ex vivo*	JM/SS	ESD-R	55	30	1
5	*Ex vivo*	SS/JM	CAP	80	71	0
6	*Ex vivo*	JM/SS	CAP	70	65	0
7	*Ex vivo*	SS/JM	ESD-R	30	14	0
8	*Ex vivo*	JM/SS	ESD-R	18	10	0
9	*Ex vivo*	SS/JM	CAP	30	25	0
10	*Ex vivo*	JM/SS	CAP	40	33	0
11	*Ex vivo*	SS/JM	ESD-R	16	8	0
12	*Ex vivo*	JM/SS	ESD-R	11	5	0
13	In vivo	SS/JM	ESD-R	56	16	1
14	In vivo	JM/SS	ESD-R	43	25	1
15	In vivo	SS/JM	ESD-R	71	23	0
16	In vivo	KS/SS	ESD-R	52	26	0
17	In vivo	JE/SS	ESD-R	38	20	0
18	In vivo	KS/JE	ESD-R	26	11	1

### In vivo ESD

**Fig. 7:**
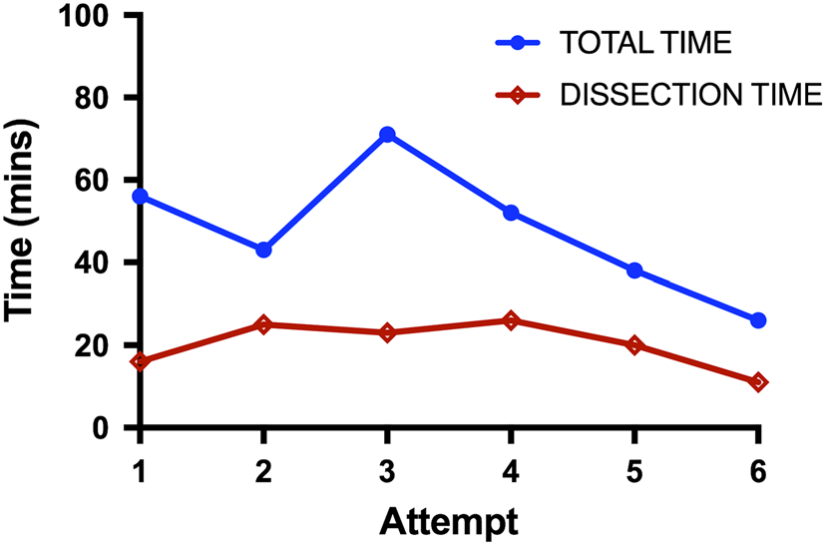
Time by attempt (in vivo ESD-R technique)

FB-clip placements were successful in all six cases. Total procedural median time for ESD-R was 48 ± 15 min, this did not significantly differ from the *ex vivo* cap or ESD-R methods (*p* = 0.2 and 0.07 respectively). However, dissection time was significantly shorter 20 ± 6 min. In addition, total time decreased significantly following six attempts (60 vs. 30 min) and the dissection time also diminished as procedure number increased (see Fig. 7). During ESD, the FB-clip traction provided the endoscopist with direct visualization of the submucosal layer, and the tension force could be precisely controlled from the control handle (Fig. 5H). There was no inadvertent tearing of the specimen. All ESDs were completed as previously planned in the *ex vivo* experiment.

The maximal diameter of specimens was 6 cm (average 5 cm [range 4–6 cm]). There were minor perforations in three of the cases, managed by clipping. After lesion removal, the specimen was removed with the endoscope from the animal as it was already clipped to the FB—acting as a specimen retrieval system (see Fig. 5I). There were no episodes requiring hemostasis.

## Discussion

The human colonic lumen remains a challenging anatomical arena to perform minimally invasive surgery. This challenge is compounded by the relatively high perforation risk [4, 10, 11] and subsequent sterile peritoneum contamination when performing advanced endoscopic procedures such as ESD. Much of this can be attributed to the relative minimal margin of safety in the thin colon wall (relative to for example the stomach) as well as the lack of available current technology in providing operative field control.

Single and double-balloon technology has been used for many years to aid small bowel enteroscopy [12]. However, recent reports describe the use of balloon systems in the lower GI tract for ESD in providing some control through stabilization, particularly in challenging anatomical configurations [13]. Deficiencies in these techniques remain, including fixed balloon position on the endoscope, optimization for upper GI applications, and use of the endoscope working channel (preventing additional tool passage).

Here, we report the first *ex vivo* and in vivo experience of a unique double-balloon endoscopic device. Its independent inflation control and distance adjustments overcome some of the challenges of endoscopy such as stabilization of the endoscope tip and improved visualization. These features are primarily added through inflation of two balloons and TZ set-up between them. The AB being fixed in position behind the endoscope tip centralizing it within the lumen and providing stabilization relative to the lesion. The fore-balloon acted as a ‘hand-retractor’ in-front of the endoscope tip which could be moved variably according to the operators preference for distance. The FB also acted as a tissue retractor through clipping of the FB to the incised lesion mucosal edge. To evaluate the value of using such a device, we used the most challenging current endoscopic therapeutic method—ESD.

EMR and ESD techniques prevail in treating polyps and early cancers of the GI tract. ESD has a strong popularity in Eastern countries such as Japan. Despite the clear benefits of ESD over EMR (en-bloc resection and lower recurrence rate), the current strain on resources obviates its justification in the West [14]. However, much of the increased procedural time can be attributed to poor stability, visualization, fear of perforation, and slow dissection progression—currently a millimeter by millimeter method facilitated by a plastic cap attached to endoscope tip.

In our early experience, the ESD-R technique did exhibit a higher minor perforation rate (one of the six in the *ex vivo* group and three of the six in the in vivo group) compared to control. These perforations were clipped closed and were deemed not clinically significant. Two reasons would explain this phenomenon (1) the ESD-R technique causes retraction of the submucosal tissues lifting the muscle layer up increasing the likelihood of inadvertent muscle damage and (2) operators are still most likely on the learning curve.

We have demonstrated that TZ set-up and FB extension provided tissue traction, and aided the mucosal dissection process. Complex polypectomy duration using the double-balloon platform was significantly shorter in our *ex vivo* work. The transference of these skills in vivo corroborated the procedural duration reduction in that the dissection time was on average 20 min. The observed average total procedural time of 48 min in the in vivo study indicated that over 50% of the time taken was setting up the tissue traction—particularly with regards to clip placement. This time was still marginally less than *ex vivo* cap ESD (not statistically significant) and longer than the *ex vivo* ESD-R (*p* = 0.07) suggesting a learning curve. By the last attempts, the total procedural time was similar to the dissection time, further suggesting a likely learning curve in transferring the skills in vivo. Our data indicated that the necessary skills might be achieved in six attempts, one limitation of this was the small number of attempts, further attempts would have been needed both *ex vivo* and in vivo to qualify the learning curve. Once FB-clip tissue traction was successful, the improved visibility and tension increased safety and reduced dissection duration significantly.

Specimen retrieval can be a challenging aspect of endolumenal procedures. Specimens can be difficult to locate once removed by the operator. Smaller specimens can occlude the endoscope working channel and larger ones difficult to manage. To address this, some solutions have been reported [7–9]. To our knowledge, this is the first report using a specially designed balloon to retrieve specimens endolumenally.

This study has several limitations, including the relatively small number of lesions tackled. We were also unable to perform a necropsy to assess the resection craters for microperforations that may not have been visible on endoscopy. We also opted not to have a control in the in vivo study due to resource constraints.

In conclusion, we have demonstrated the development of a unique technical method for ESD through *ex* and in vivo means and demonstrated its superiority over conventional *ex vivo* ESD methods. This was mediated by a double-balloon device providing increased stability, improved visualization and tissue traction, which significantly reduced dissection time. In addition, specimen retrieval capabilities provided a safe method to retrieve specimens and under direct vision. Such an approach may reduce institutional resource pressures and pave the way for further endolumenal procedure development.

## Electronic supplementary material

Below is the link to the electronic supplementary material.

Supplementary Video 1 (MP4 31865 KB)
